# COMSOL-Based Modeling and Simulation of SnO_2_/rGO Gas Sensor for Detection of NO_2_

**DOI:** 10.1038/s41598-018-20501-2

**Published:** 2018-02-01

**Authors:** Farshad Yaghouti Niyat, M. H. Shahrokh Abadi

**Affiliations:** Electrical and Computer Engineering Faculty, Hakim Sabzevari University, Sabzevar, 9617976487 Iran

## Abstract

Despite SIESTA and COMSOL being increasingly used for the simulation of the sensing mechanism in the gas sensors, there are no modeling and simulation reports in literature for detection of NO_2_ based rGO/SnO_2_ sensors. In the present study, we model, simulate, and characterize an NO_2_ based rGO/SnO_2_ gas sensor using COMSOL by solving the Poisson’s equations under associated boundary conditions of mass, heat and electrical transitions. To perform the simulation, we use an exposure model for presenting the required NO_2_, a heat transfer model to obtain a reaction temperature, and an electrical model to characterize the sensor’s response in the presence of the gas. We characterize the sensor’s response in the presence of different concentrations of NO_2_ at different working temperatures and compare the results with the experimental data, reported by Zhang *et al*. The results from the simulated sensor show a good agreement with the real sensor with some inconsistencies due to differences between the practical conditions in the real chamber and applied conditions to the analytical equations. The results also show that the method can be used to define and predict the behavior of the rGO-based gas sensors before undergoing the fabrication process.

## Introduction

In the recent years, many scientists have focused on the pollution of environment and its consequences such as global warming. The attention is mainly due to the problems related to the release of a large amount of toxin gases such as Nitrogen Dioxide (NO_2_), induced by imperfect combustion in automobiles and industrial zones^1^. Different methods from sophisticated analytical lab and gas chromatography systems to miniaturized hand-held devices have been introduced and used for detection of particular amount of NO_2_. Among the hand-held sensors, Metal-Oxide Semiconductor (MOS) gas sensors have greatly attracted the researchers attentions for detection of various types of gases due to their prominent quality of high sensitivity, high selectivity, low power consumption, fabrication simplicity, low cost, etc.^2^. The performance of such sensors can be promoted by adding special amount of additives and catalysts to the MOS materials to make them highly selective to a specific target gas, increase their sensitivities, and reduce the operating temperature, which is a necessary parameter for reducing the power consumption and to give a minimum activation energy, *E*_*A*_, to the gas molecules, experiencing an optimum reaction. Furthermore, reducing the size of MOS and catalyst particles to nano scales is resulted in greater surface-area-to-volume ratio, which in turn gives more available reaction sites for the molecules of the target gas.

Tin oxide, SnO_2_, is a well-known *n*-type MOS material, which has been intensively studied with various shapes and morphologies along with different types of catalysts and dopants for detection of variety gases. Recently, graphene, built from the 2D array of atoms jointed together with covalent sp^2^ bonds, and its derivative oxides including graphene oxide (GO) and reduced graphene oxide (rGO), has caught the attention of many researchers due to its advantages as a catalyst within SnO_2_ phases that can help activating reactions occurring on the carbon surface by favoring the adsorption/desorption processes^3^. Graphene possesses high mobility carriers and the fact that the adsorption of gas molecules can alter this mobility and its associated electrical properties, so that graphene assisted tin oxide gas sensors show high sensitivity to low concentrations of NO_2_^4,5^.

One of the challenges that limits the performance of NO_2_ gas sensors based on SnO_2_/graphene is their high operating temperature. To overcome this problem, rGO with high electron mobility at room temperature has been used as a next promising sensing material for detection of NO_2_ at lower temperatures, the fact that can be explained based on generously adsorption/desorption on the C-sites because of the critical role played by *n*-SnO_2_/*p*-rGO heterojunction formed on the composite materials^5–10^.

Fabrication of a new MOS/rGO nanocomposite gas sensor always faces with many challenges including time, budget, and a better understanding of the sensing mechanisms and consequences resulting from the reaction with the target gas. One way to reduce the expenditures is to predict the sensing behavior of the sensor before the fabrication^11^. In this paper, a fabricated SnO_2_/rGO NO_2_ sensor, reported by Zhang *et al*.^12^, having the same structure, size, and materials has been modeled and simulated by solving the associated equations using COMSOL Multiphysics 5.2a software. The simulated results show a very close match compared with the experimental results, proving that the method can be widely extended and applied to other nanocomposite-based gas sensors.

## Results and Discussion

The simulation results of the sensor consist of three basic models; the NO_2_ gas exposure model, the sensor heat transfer model, and the extracted electrical properties model of the sensor, which are considered and discussed in the following subsections.

### The NO_2_ Exposure Model

**Figure 1:**
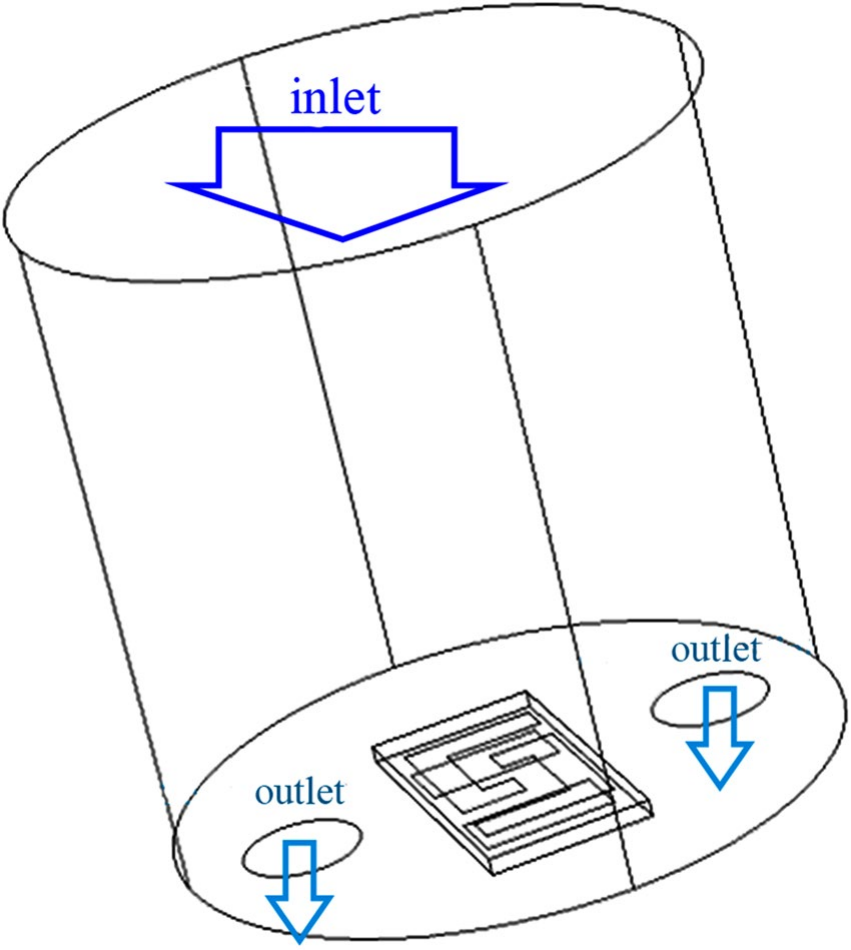
The chamber for exposing the sensor to the applied gas.

**Figure 2:**
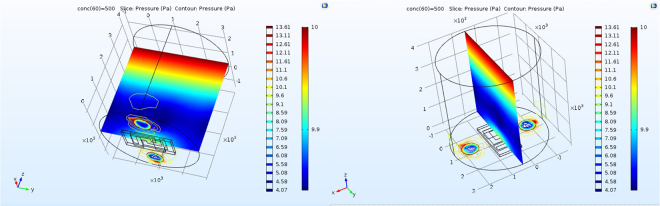
Distribution of the applied gas passing through the chamber from the top to the bottom base.

In order to apply NO_2_ to the sensor, a cylindrical chamber (*d* = *h* = 4 mm) with an open base as the inlet and two holes (*d* = 0.35 mm) as the outlets, shown in Fig. 1, was designed and the sensor was located between the holes with an upward active area. The gas is applied from the upper base of the chamber and collected from the outlet holes with an illustrated pressure distribution given in Fig. 2. This model simulates the flow of the gas inside the chamber and it can be used for variety gases at different concentrations.

### The Heat Transfer Model

**Figure 3:**
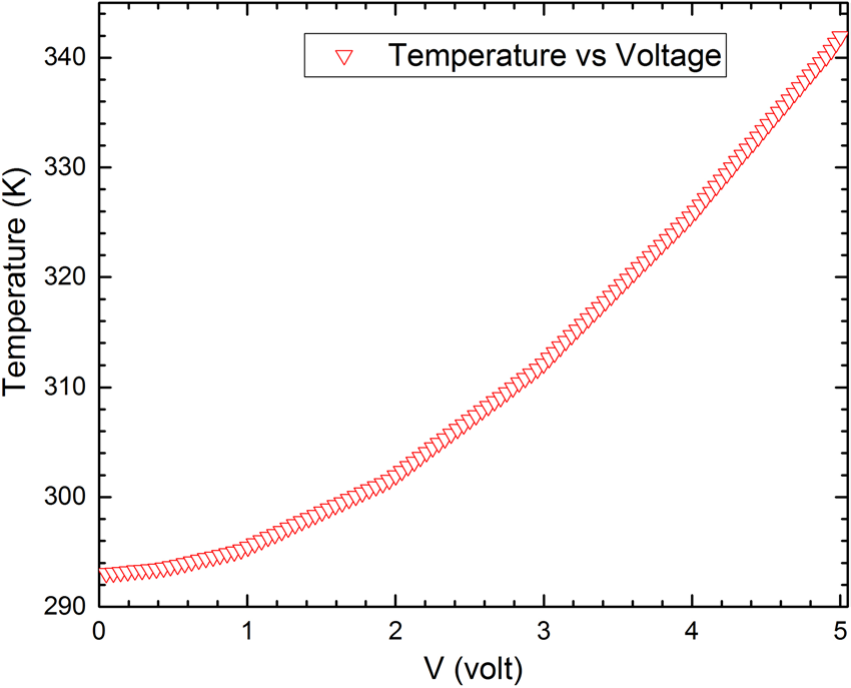
The temperature-voltage curve of the sensor.

**Figure 4:**
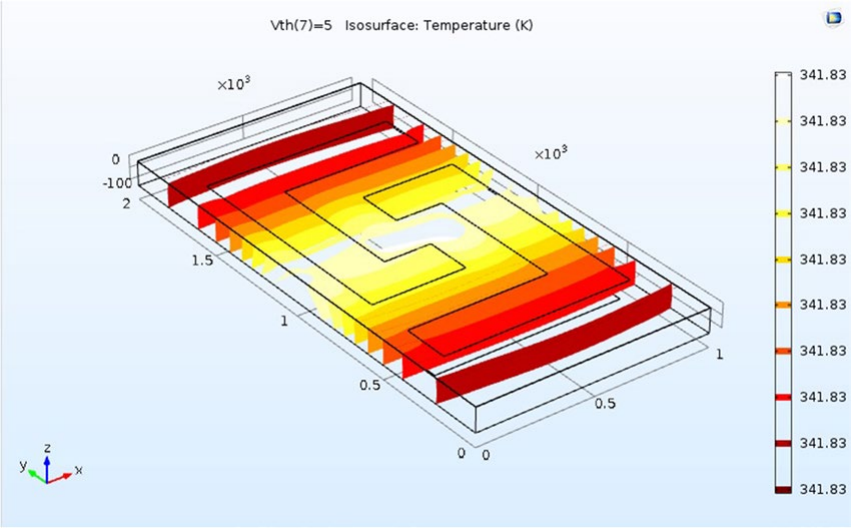
The temperature distribution on the surface of the sensor shows very small temperature variations, less than 0.001 K.

To model the heat transferring, the heater was supplied with a DC voltage ranging between 0.1 to 5 volts and simultaneously the variations of the temperature on the surface was recorded. The relation between the applied voltage and increasing the temperature of the surface has been shown in Fig. 3. The temperature of the sensor will reach the room temperature (RT = 300 K) at voltages around 1.5 V and increase to 343 K at 5 V. Figure 4 shows the heat dispersion over the surface of the sensor supplied at 5 V. Due to the high thermal conductivity of the substrate and the small size of the sensor, the surface of the active layer becomes isothermal with a low thermal variation, and gives an excellent uniformity of the heat distribution on the active layer with the variations in the order of 10^−4^/K, an advantageous parameter for sensing applications. Afterwards, this model must be accompanied by the subsequent models to obtain the electrical properties of the sensor.

### The Time-Resistance Characteristic of the Sensor

**Figure 5:**
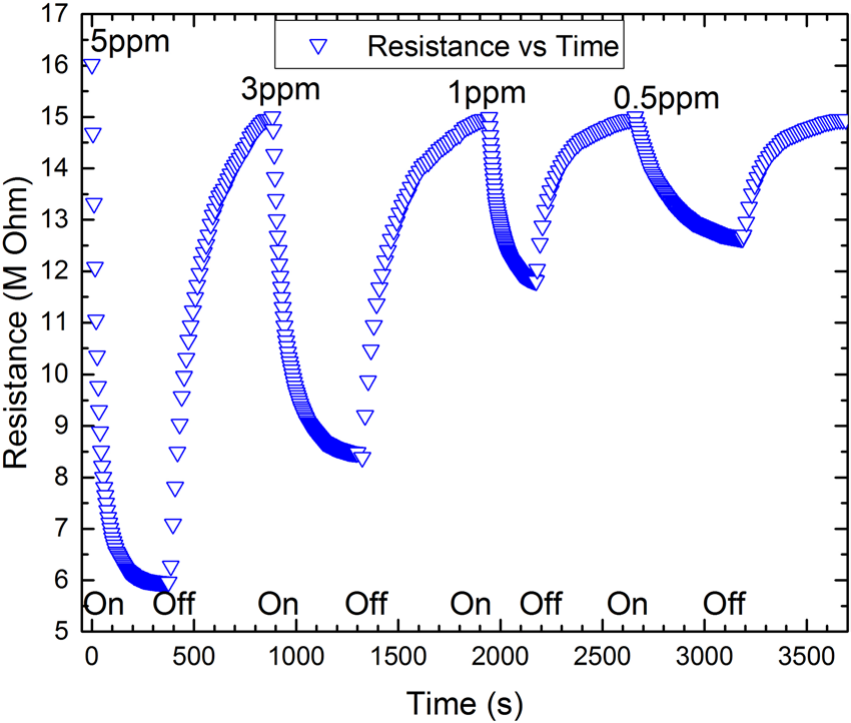
The time-resistance curve of the simulated sensor working at 50 °C.

The time-resistance relation of the sensor was simulated at 5, 3, 1 and 0.5 ppm of the NO_2_ at working temperature of 50 °C, shown in Fig. 5. A rapid change in the curves is observed at the time of exposure to the gas, then the response reaches a steady state, and the same phenomenon occurs when the gas is removed from the chamber and fresh air is applied, which is in accordance with the numerical solution of the Navier-stokes equation for the field velocity of the gas, that will be discussed later in the equation (3). The data for the response and recovery times were extracted from the COMSOL and analyzed in the OriginPro 2016 Software, where the times have been found to be about 148 s and 225 s at 5 ppm NO_2_, respectively. Lowering the concentration of the applied gas will reduce the changes of the sensor’s resistance.

During the exposure to the NO_2_, the reduction of the resistance indicates that the charge carriers of the composite are mostly holes, demonstrating the p-type essence of the rGO. In such an environment, an adsorbed NO_2_ molecule acts as an electron-donor that stimulates the charge carrier concentration in the graphene sheet. As the main role for the nanocomposites sensing mechanism, the bandgap of the rGO is modulated and results in the reduction of the sensor’s resistance^3,13^. The modulation, in fact, is influenced by the p-n heterojunction which is formed between p-type rGO and n-type SnO_2_. Since the rGO is the major component in the composite, acts as an electron-absorption body and due to its higher work function (∑4.75 eV) in compared with the SnO_2_ (∑4.55 eV), it receives electrons from the neighbor SnO_2_ and causes the resistance modulation^14^.

When the flow of NO_2_ is turned off and the fresh air is applied, the initial resistance of the sensor is reestablished and the sensor’s resistance is retained, starting the desorption mechanism of the NO_2_ molecules from the sensor’s surface^13,14^.

### The *R*_*a*_/*R*_*g*_-time Characteristic of the Sensor

**Figure 6:**
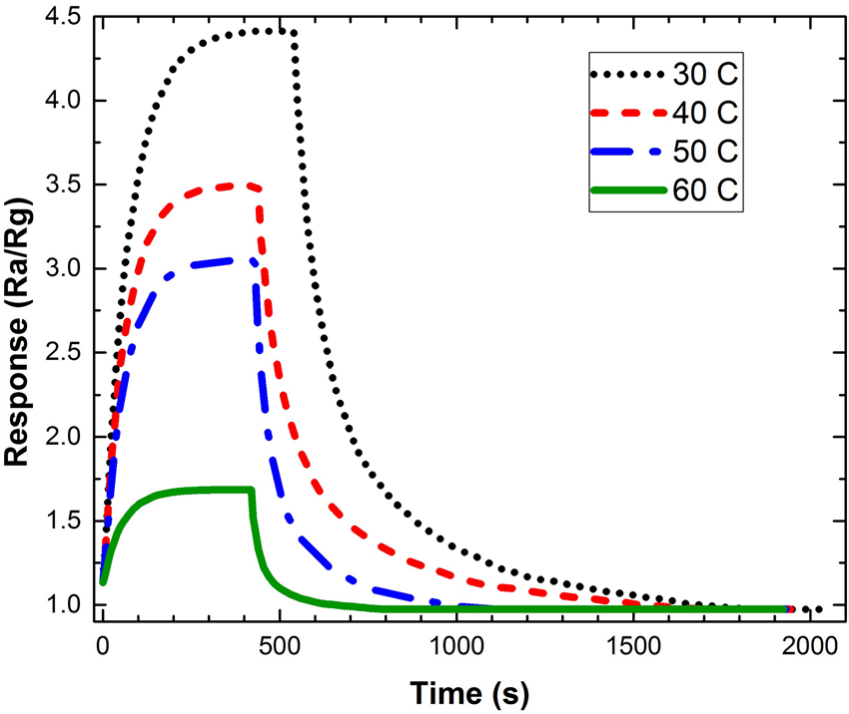
The simulated results of response-time to 5 ppm NO_2_ at 30, 40, 50, and 60 °C working temperatures.

The response of the sensor can be characterized as the ratio of the sensor’s resistance in the air to the resistance in the presence of the gas, *R*_*a*_/*R*_*g*_, versus the time. Figure 6 shows the response of the sensor exposed to 5 ppm NO_2_ at 30, 40, 50, and 60 °C working temperatures. Due to the dominating of rGO, during the exposure time the resistance of the sensor shows a temperature dependency over the temperature range, as shown in Fig. 6. Generally, the electrical conductivity of the rGO sheets depends exponentially on the temperature (*σ* ~ exp(−*T*^−1/2^))^14,15^. The phenomenon is explained by the Efros–Shklovskii Variable-Range Hopping (ES-VRH) mechanism, which deals with electrical conductivity as a function of temperature for disordered systems. The generalized form of electrical resistance based on VRH is described by

1 $${R}({T})={{R}}_{0}\exp {(\frac{{{T}}_{0}}{{T}})}^{{m}}$$

where *T*_*0*_ is the characteristic temperature and the exponent *m* = 1 corresponds to thermally activated process, *m* = 1/2 corresponds to ES-VRH, or m can be 1/3 or 1/4 corresponding to 2D-VRH or 3D-VRH model, respectively^16^. Therefore, increasing the temperature will reduce the resistance of the contact points in rGO to its initial value of the *R*_*0*_. Meanwhile, if the NO_2_ is replaced with the fresh air, the resistance of the sensor goes back to about its initial value, known as recovery state, which has been already shown in Fig. 5. It is also clear for achieving faster recovery time the heater should be biased at higher voltages.

### The Response-Concentration Characteristic of the Sensor

**Figure 7:**
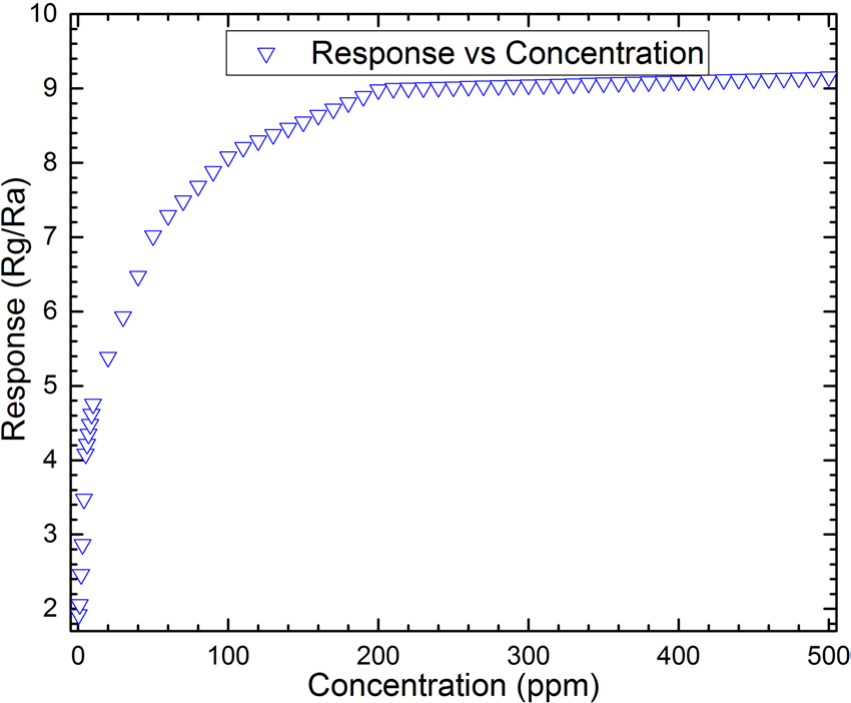
The response-concentration curve of the simulated sensor exposed to 0 to 500 ppm NO_2_ working at 50 °C.

As it has been already shown, at higher concentrations of NO_2_, the sensor’s resistance is decreased, which causes an increasing in *R*_*a*_/*R*_*g*_. This phenomenon attributes to increase of the electron density associated with the numbers of NO_2_ molecules, resulted in decreasing the sensor resistance. Figure 7 shows the simulated results of the sensor’s response from 0 to 500 ppm NO_2_. As it can be seen from the figure, the concentrations above 200 ppm do not have a significant impact on the sensor’s response, showing that the active vacancy sites of SnO_2_/rGO film are rapidly filled and saturated by either O^−^ or O^−2^ ions at lower concentrations. An expansion for the current density, *J*, based-on the number of occupied sites by the oxygen ions in the Poisson’s Equation, given in the equation (12) in the following subsections, is the basis of the analytical approach in the COMSOL software for the results given in the Fig. 7.

### Data Validity Correlation and Confirmation

Since the experimentation consists of a series of test executions under physical and environmental conditions, its results are real by definition comparing to the simulation results. Nonetheless, because of the great influence of the flow of the gas in the test chamber, dissimilarities in the heat distribution on the surface of the sensor, nonequidistant arrangement of coated materials, etc.; the experimental results are hard to reproduce by the simulation. Clearly, each fabricated sensor has its own relative merits; none can be applied equally well to all aspects of the simulated model.

**Figure 8:**
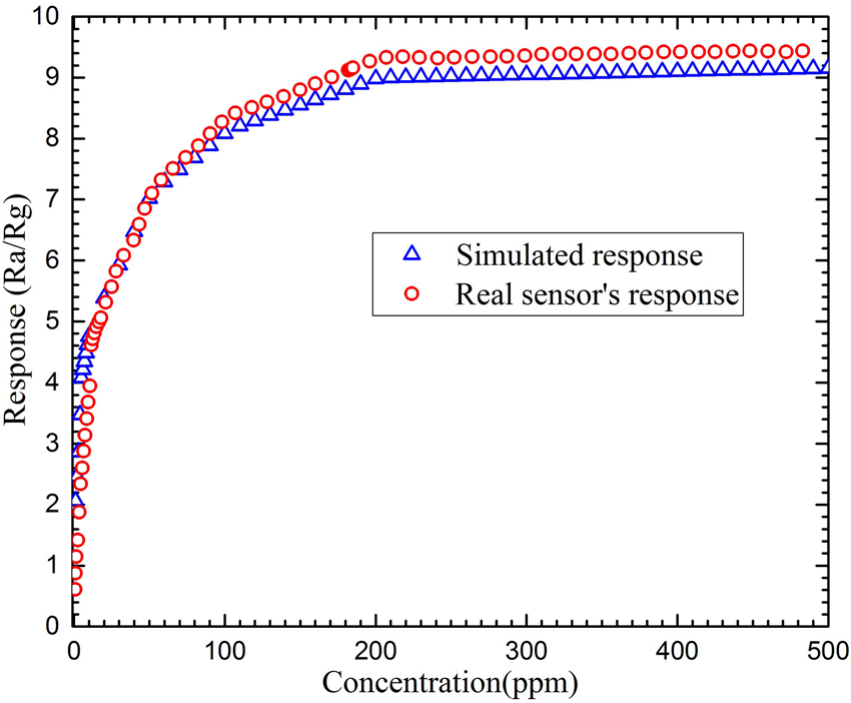
The response-concentration graph of the real sensor vs. the simulated results.

**Figure 9:**
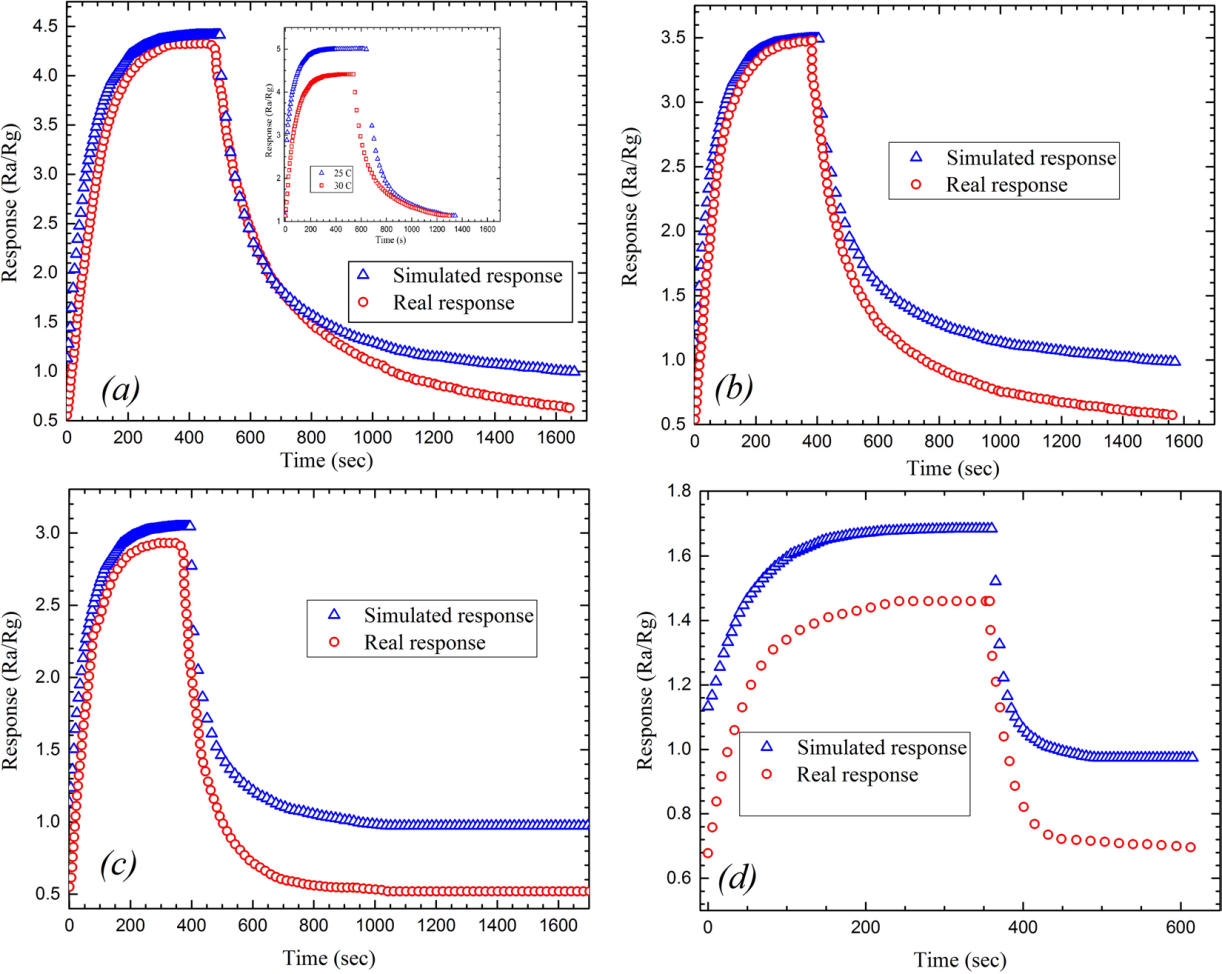
The response‒recovery‒time graphs of the simulated and fabricated sensors exposed to 5 ppm NO_2_ at different operating temperatures (**a**) 30, (**b**) 40, (**c**) 50, and (**d**) 60 °C. Inset in (**a**) compares the response-recovery-time graphs of the simulated sensor working at 25 °C versus the response of the real sensor working at 30 °C.

Figure 8 shows the sensitivity of the sensor at different concentrations of NO_2_ for both the fabricated and simulated sensors with a greater response at concentrations above 100 ppm for the real sensor. The response‒recovery‒time of the sensors exposed to 5 ppm NO_2_, working at different operating temperatures, also has been shown in Fig. 9.

Although the experimental and simulated data are in reasonable agreement with respect to the shape of the graphs and the location of the maxima (see Figs 8 and 9 except for Fig. 9d), showing almost the same features at different operating temperatures and gas concentrations, there are some contradictions between the results that should be explained. From Fig. 9, the best fit for the graphs is observed for the working temperatures less than 40 °C. As the temperature is increased the response-recovery-time graphs undergo two types of change: first, the recovery time goes far from the experimental values, second, the level of the maxima is altered. The latter can be observed in Fig. 9d while the former has been depicted in Fig. 9b and c. While there is a great similarity in the responses of simulated and fabricated sensors to the gas concentrations less than 100 ppm (Fig. 8), the uncorrelated recovery-times may be attributed to increasing the operating temperature. Therefore, more efforts need to take into account for making a complete compatibility between the simulation and experimental results for both sensors working at same temperature.

The inset in Fig. 9a compares the response of the simulated sensor working at 25 °C and response of the real sensor working at 30 °C. It is notable to mention that we didn’t have any data for the response of the real sensor at this operating temperature for further comparison. It can be seen that the maximum peak of the response has been increased as the working temperature decreases, whilst the recovery curve of the simulated sensor follows the recovery curve of the real one.

It is known that the response of rGO and SnO_2_ for the gas sensing applications depends to the working temperature, while the SnO_2_ gas sensor usually works at high temperatures, the rGO alone responds to the target gases efficiently at ambient temperatures. The presence of rGO in the SnO_2_–rGO nanocomposite causes the optimum operating temperature lays between that of rGO and SnO_2_ and since the rGO is the major component in the composition, the optimum temperature gets closer to the room temperature. This is attributed to the dependency of thermal conductivity of rGO which follows Efros-Shklovskii variable range hopping conduction in the room temperature or less. Each rGO molecule undergoes to an oxidation reaction during heat alteration which cannot be easily simulated. Therefore, it can be concluded that the main difference between the simulated graphs of the response-recovery time may be attributed to the differences in thermal flux density between the experimental and the simulation environment, as it has been described later in Heat Transferring subsection. In order to obtain an accurate estimation of the impact of the thermal flux on the simulation results it is necessary to measure the temperature distribution on the surface of the real sensor using an array of temperature sensors.

Furthermore, the inconsistencies that are seen in the responses between the fabricated sensor and the simulated one can be interpreted due to the experimental conditions such as the effects of concentration variations of NO_2_ on the surface of the active layer, the temperature alterations on the active layer, as well as the mesh structure used for the simulation.

## Method: Approaching to the Analytical Model

For simulation of the MOS gas sensors, usually four physics; solid mechanics, electrostatics, electric current and heat transfer physics are involved and are sequentially solved in the 2D platform. Generally, in the MOS gas sensors, three major processes are taken into account to describe the whole procedure of gas sensing: (1) the operating temperature and heat transfer from the heater of the sensor, where the best response can be observed, (2) the exposure of the sensor’s active area to the target gas, (3) the electrical response due to the reaction between NO_2_ molecules and the active layer. In the following subsections the equations that describe the aforementioned processes are briefly explained.

### Exposing the Active Layer to NO_2_

The flow of the gas in the cylindrical chamber is described by Navier-stokes equation, given by

2 $$\rho \frac{D\vec{U}}{Dt}=-\nabla P+\rho g+\eta {\nabla }^{2}U$$

where *ρ*, *U*, *P*, *g*, and *η* are gas flux, velocity field, gas pressure, gravity, and viscosity, respectively. The equation can be developed into Cartesian coordination as follows

3 $$\{\begin{array}{c}P(\frac{\partial u}{\partial t}+{\boldsymbol{u}}\frac{\partial u}{\partial x}+{\boldsymbol{v}}\frac{\partial u}{\partial y}+{\boldsymbol{w}}\frac{\partial u}{\partial z})=-\frac{\partial \rho }{\partial x}+\mu (\frac{{\partial }^{2}u}{\partial {x}^{2}}+\frac{{\partial }^{2}u}{\partial {y}^{2}}+\frac{{\partial }^{2}u}{\partial {z}^{2}})+\rho {g}_{x}\\ P(\frac{\partial v}{\partial t}+{\boldsymbol{u}}\frac{\partial v}{\partial x}+{\boldsymbol{v}}\frac{\partial v}{\partial y}+{\boldsymbol{w}}\frac{\partial v}{\partial z})=-\frac{\partial \rho }{\partial y}+\mu (\frac{{\partial }^{2}v}{\partial {x}^{2}}+\frac{{\partial }^{2}v}{\partial {y}^{2}}+\frac{{\partial }^{2}v}{\partial {z}^{2}})+\rho {g}_{y}\\ P(\frac{\partial w}{\partial t}+{\boldsymbol{u}}\frac{\partial w}{\partial x}+{\boldsymbol{v}}\frac{\partial w}{\partial y}+{\boldsymbol{w}}\frac{\partial w}{\partial z})=-\frac{\partial \rho }{\partial z}+\mu (\frac{{\partial }^{2}w}{\partial {x}^{2}}+\frac{{\partial }^{2}w}{\partial {y}^{2}}+\frac{{\partial }^{2}w}{\partial {z}^{2}})+\rho {g}_{z}\end{array}$$

where ***u***, ***v***, and ***w*** are the velocity in direction of *x*-, *y*- and *z*-axis, respectively. To achieve the field velocity of the gas, Navier-stocks equations must be simultaneously solved using the continuity equation given by

4 $$\frac{\partial \rho }{\partial t}+\nabla ({\boldsymbol{\rho }}\vec{{\boldsymbol{U}}})=0$$

Since the gas flow is incompressible, *ρ* becomes constant, therefore the equation is reduced to

5 $$\nabla \vec{{\boldsymbol{U}}}=0$$

### Heat Transferring

The amount of heat transferred by a heater to its environment is determined by either its thermal conduction, convection, or radiation. The heat from the heater of a gas sensor to its active layer is transferred by thermal conduction. Thermal conduction, also known as thermal diffusion, is defined as the heat transfer occurred by collisions of the particles and electron’s movement in a body. The Fourier’s law, which states that the negative gradient in the temperature is proportional to the heat transfer in a material, is the law that governs the thermal conduction given by

6 $$\mathop{q^{\prime\prime} }\limits^{\longrightarrow}=-k\overrightarrow{\nabla T}$$

where *q*″, *k*, and *T*, are flux density, conductivity of material, and temperature, respectively. The Fourier**’**s law, then, can be written as

7 $$q^{\prime\prime} =-k({\boldsymbol{i}}\frac{dT}{dx}+{\boldsymbol{j}}\frac{dT}{dy}+{\boldsymbol{k}}\frac{dT}{dz})$$

Therefore, one can write the heat flux in the direction of *n* as

8 $${q^{\prime\prime} }_{n}=-k\frac{dT}{dn}$$

So the total heat flux from Fourier’s law can be rewritten as

9 $$q^{\prime\prime} ={\boldsymbol{i}}{q^{\prime\prime} }_{x}+{\boldsymbol{j}}{q^{\prime\prime} }_{y}+{\boldsymbol{k}}{q^{\prime\prime} }_{z}$$

In addition to the thermal conduction, portions of the heat are transferred into the air and to the edges of the device by convection and radiation, either by heater or by active layer itself. In thermal convection, the movement of heat occurs by the mass transferring from one place to another. The basic relationship in heat transfer convection is described by Neumann’s law of cooling

10 $$q=q^{\prime\prime} A=hA({T}_{a}-{T}_{b})$$

where *q*, *q*″, *h*, *A*, *T*_*a*_, and *T*_*b*_ are the heat transfer, heat flux, heat transfer coefficient, surface area, surface temperature, and air temperature, respectively. In the thermal radiation, heat travels by photons in form of electromagnetic waves and emits energy. The rate of radiant heat is described by Stefan-Boltzmann equation, given as

11 $$q=q^{\prime\prime} A=\varepsilon \sigma A{T}_{s}^{4}$$

where *q*, *q*″, *A*, *ε*, *σ*, and *T*_*s*_ are heat transfer, heat flux, surface area, emissivity coefficient, Stefan-Boltzmann constant, and surface temperature, respectively.

### Electrical Statements for the Active Layer

When the active layer is exposed to the NO_2_ molecules, either a surface, a bulk, or a combination of both reactions would happen, which results in a change of the sensor’s response. The interaction between the NO_2_ molecules and the active layer can be described using equation (12) as the basis of Poisson’s equation

12 $$\{\begin{array}{c}\nabla J=0\\ J=\sigma \xi \\ \xi =-\nabla V\end{array}$$

where *J*, *ξ*, and *V* are current density, electrical field, and electrical potential, respectively. Then Poisson**’**s equation is obtained as

13 $$-{\nabla }^{2}V=0$$

Whichever reaction takes place in the active layer, COMSOL software uses Poisson’s Equation given by equation (13), to construct the electrical parameters of the sensor, such as current, resistance, and their associated responses.

### Boundary Conditions

In the most cases, the boundary conditions for simulation a MOS gas sensor are fixed type at the bottom layer of the heater and flexible at the top of active layer. In this work, the boundary conditions of the device are applied in three different domains for solving the mass transfer equations, the heat transfer equations, and the electrical equations, given in the following subsections.

#### The boundary conditions for the NO_2_ exposure

Since there is no slip on the wall-side in the chamber, the boundary condition due to the slip is described as

14 $${U}_{T}=0$$

where *U*_*T*_ is a tangential velocity.

In addition, the boundary conditions due to the pressure of inlet and outlet gas respect to the chamber are defined as

15 $$\{\begin{array}{c}{P}_{inlet}=0\\ {P}_{outlet}=0\end{array}$$

#### The boundary conditions for the heat transfer

During the heat transfer, the boundary conditions are applied to both the convection and radiation components. The boundary conditions in the heat transfer convection are described by Neumann equations, given by

16 $$\{\begin{array}{c}-k\frac{\partial T(0,t)}{\partial x}=hA[{T}_{\infty }-T(0,t)]\\ -k\frac{\partial T(1000,t)}{\partial x}=hA[{T}_{\infty }-T(1000,t)]\end{array}$$

where *T*_*∞*_ is considered as room temperature (300 K) and *T*(0, *t*) and *T*(1000, *t*) are the temperature sides of the sensor. The boundary conditions for the heat transfer radiation are described using Stefan-Boltzmann equations:

17 $$\{\begin{array}{c}-k\frac{\partial T(0,t)}{\partial x}=\varepsilon \sigma A[{T}_{\infty }^{4}-T(0,t)]\\ -k\frac{\partial T(1000,t)}{\partial x}=\varepsilon \sigma A[{T}_{\infty }^{4}-T(1000,t)]\end{array}$$

with the same parameter described for equations (10), (11) and (16).

#### The electrical boundary conditions for the active layer

The active layer of the sensor is electrically insulated from the environment, therefore the boundary condition for the current density of the sensor can be described as

18 $$n.J=0$$

where *n* is the normal vector of plane of active layer from both sides and *J* is current density.

#### The novelty of the work and the simulation process

Based on the literature, there is no any related work that has simulated an NO_2_ based rGO-SnO_2_ gas sensor. Normally a combination results from SIESTA and COMSOL are used to simulate a chemical reaction performing electronic structure calculations and molecular dynamics simulations in the solids. Neither SIESTA nor COMSOL has been used to simulate the rGO-SnO_2_ gas sensor. The simulation process in these software is time-independent and performed in steady state conditions, despite the fact that the response of the gas sensors is time dependent. Also, the output of SIESTA is variations of energy in the active layer, which cannot be easily translated into the electrical characteristics.

Therefore, instead of using a chemical approach to simulate the active layer, an innovative method was used to simulate the active layer of the sensor mathematically. To do so, a mathematical transfer function was extracted and constructed based on temperature, gas concentration, and time variations of the real sensor’s responses. The function then was used to characterize the active layer in the COMSOL environment. By simulating the flow of the gas and heat transfer inside the chamber, a relation between gas concentration and temperature with the electrical parameters of the simulated sensor were achieved. Subsequently, the relation was used as the input of the transfer function to obtain the electrical characteristics of the sensor by solving the Poisson’s equation in COMSOL.

To achieve an electrical response based on concentration, temperature, and time variations, a separable mathematical function, *S*, based on the aforementioned parameters was constructed as

19 $${S}({conc}.,{T},{t})={{S}}_{{1}}({conc}.)\times {{S}}_{{2}}({T})\times {{S}}_{{3}}({t})$$

**Figure 10:**
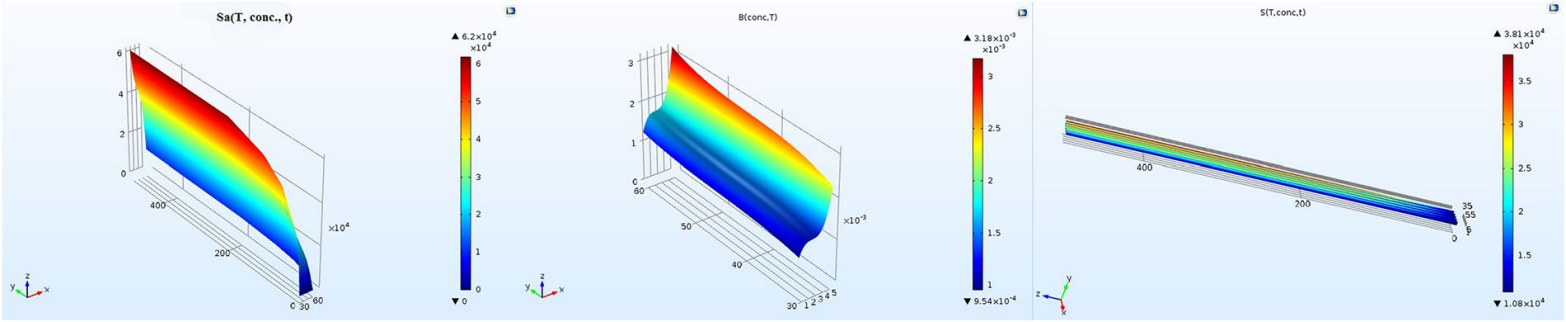
(**a**) The characteristic function of *S*_*a*_, (**b**) the characteristic function of *S*_*b*_, and (**c**) the transfer function of *S*.

**Figure 11:**
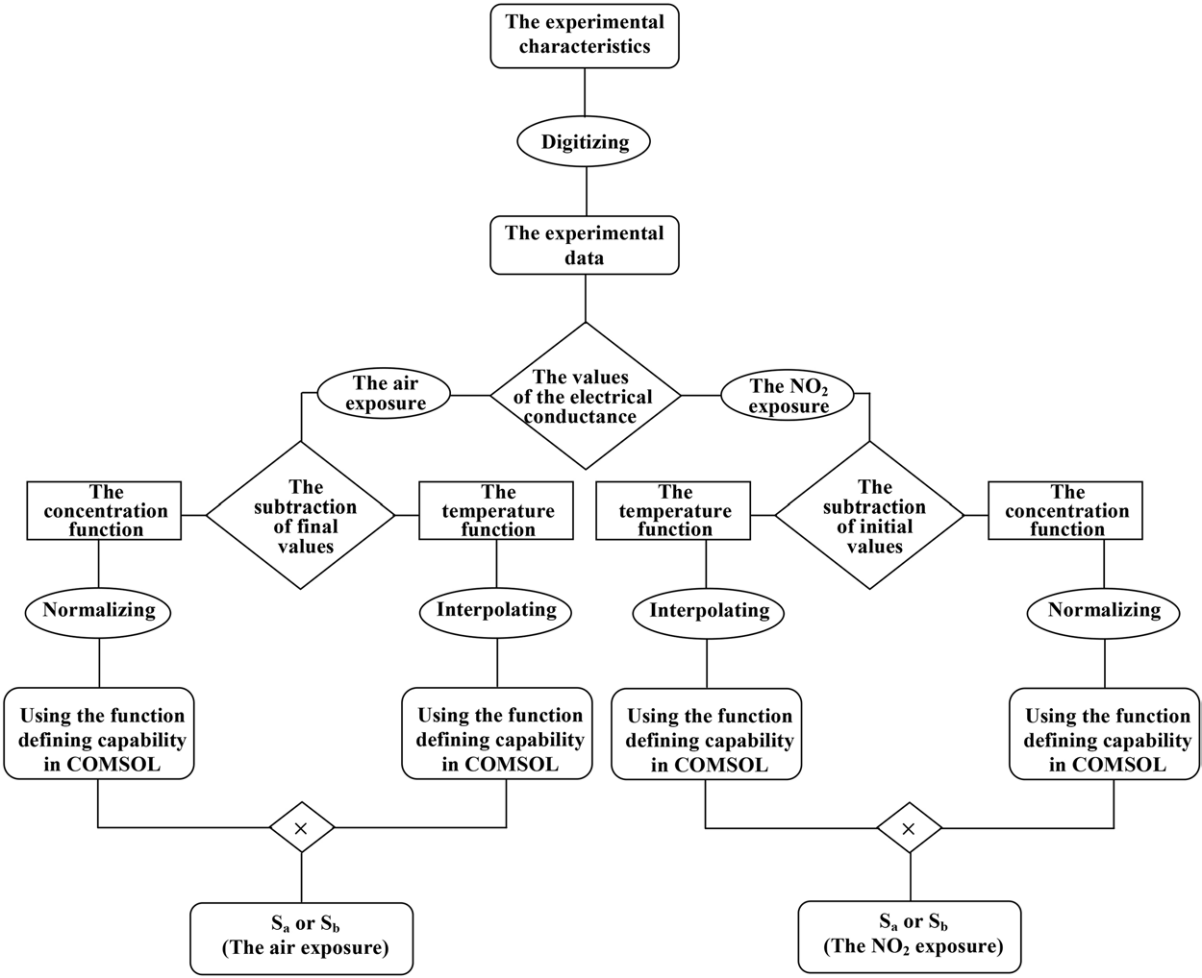
The flowchart for constructing the transfer function in COMSOL.

Using the experimental data from the original paper^12^, *S*_1_, *S*_2_, and *S*_3_ were determined by the numerical methods in MATLAB environment. Referring to the equation (10) and using the experimental data^12^, the *S* function is defined as

20 $${S}({conc}.,{T},{t})={{S}}_{{0}}+{{S}}_{{a}}({conc}.,{T})\times {{e}}^{[{Sb}({conc}.,{T})\times {t}]}$$

where the *S*_0_ is the base-line in each successful gas exposure. The exponential function multiplies by the *S*_*a*_, a function of the concentration and temperature parameters. The argument of the exponential function is time multiplied by the *S*_*b*_, the second function of temperature and gas concentration. Both the *S*_*a*_ and *S*_*b*_ can be determined from the response-time, resistance-time, and the response-concentration characteristics, to calculate the transfer function, *S*. Fig. 10 shows the simulation results for determination of each initial function and the transfer function, respectively. By convolving the parallel planes at the boundary conditions, the time-concentration-temperature variable transfer function, *S*, is defined to construct the electrical characteristics of the sensor by solving the Poisson’s equation. Figure 11 shows the steps to construct the functions that were already described.

To construct the analytical model of the active layer, data from the resistance-time, response-time, and response-concentration characteristics are extracted using a Graph-Digitizer Software. Then the alteration in the resistance of the sensor during a response to the gas can be changed to a mathematical formula, lead us to an electrical conduction statement. Also the same strategy can be taken into account when the sensor is exposed to the fresh air. In our case, those values from the electrical conduction statements are subtracted from the initial value of *S*_*0*_ (base line). At the second stage, values of the electrical conduction are subtracted from the final value to construct the *S*_*a*_ and *S*_*b*_, which are functions of concentration and temperature. Each parameter then is translated into two components: a concentration dependence and a temperature dependence, with one normalized component (here the concentration) to make the shape and the other component to function as the amplitude. Each time the sensor is exposed to the gas, the values of the normalized *S*_*a1*_ are extracted from the response curve (Fig. 5a and b in ref.^12^) while the values for the *S*_*a2*_ are extracted from the temperature curve (Fig. 5c in ref.^12^). By using the interpolation field of the COMSOL, where the values can be entered, the *S*_*a*_ function (*S*_*a1*_ × *S*_*a2*_ = *S*_*a*_) is defined with direct interpolation. Same procedure can be applied to extract the values of *S*_*b*_. Finally, the transfer function of *S* can be determined using equation (20).

### Model of the Sensor in the COMSOL Environment

#### Structure of the Model

Modeling and simulation of the sensor depends on the various parameters such as the structure and the shape, the geometry and the feature sizes, the feature mesh, and the chosen materials. For the optimization purposes, the parameters are needed to be verified and improved to obtain the maximum validity of the sensor’s response and to achieve the desired specification.

**Figure 12:**
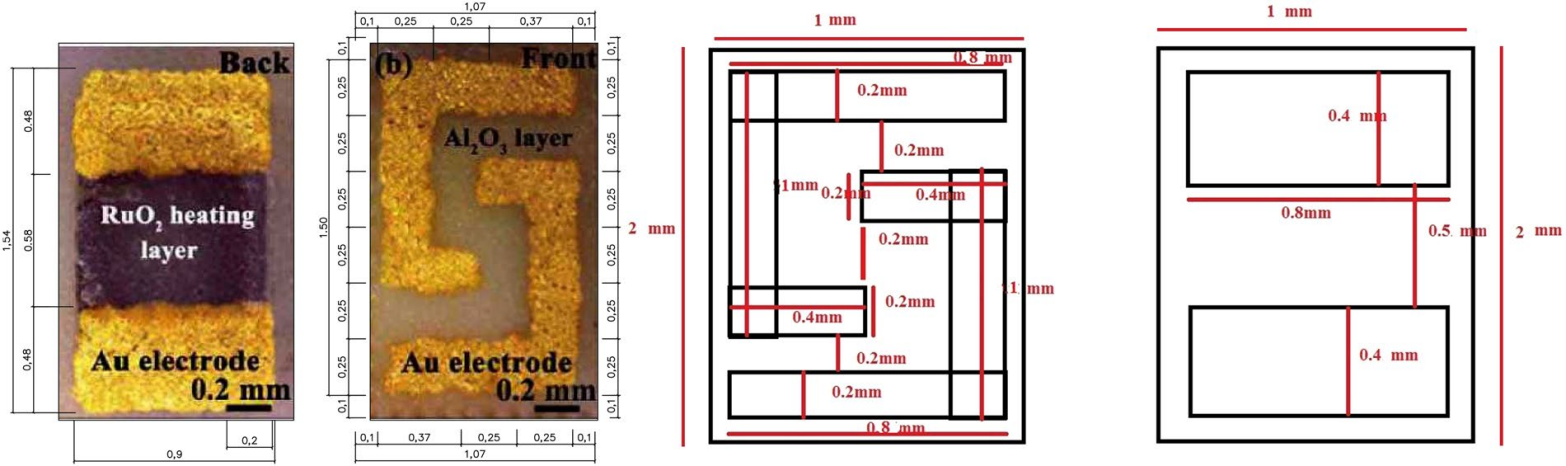
(**a**) The dimensions of the real sensor for both front and back sides and (**b**) the dimensions used for the simulated sensor.

The dimensions used for the simulation were extracted from the picture of the real sensor, given in the original paper, by using AutoCAD software. Figure 12 shows the dimensions of the real sensor versus the dimensions that have been used for the simulated sensor. It is assumed that the sensing electrodes and the meander of the real sensor are symmetric in the masking process and the inconsistencies in the film dimensions are probably due to the practical deposition during the fabrication process.

Structurally, the sensor consists of four parts: an active layer, electrodes, a meander type heater, and an alumina substrate. The following subsections are dealt with introducing these parts and their associated parameters.

#### The active layer

The active layer of the real sensor is composed of 1 µm thick nanoparticles of SnO_2_/rGO, developed in an area of almost 600 × 1400 µm^2^. To model the active layer of the sensor in the COMSOL environment based on the predefined model and the associated boundary conditions, the parameters given in the Table 1 have been used.

**Table 1:** Active layer parameters^12,17–20^.

width (μm)	length (μm)	thickness (μm)	Thermal conductivity ${\boldsymbol{w}}{\boldsymbol{/}}({\boldsymbol{m}}{\boldsymbol{.}}{\boldsymbol{K}})$	Density $({\boldsymbol{k}}{\boldsymbol{g}}{\boldsymbol{/}}{{\boldsymbol{m}}}^{{\boldsymbol{3}}})$	Heat capacity at constant pressure *J*/(*kg*.*K*)
600	1400	1	3250	1910	120

#### The electrodes

Gold, with the detailed properties presented in Table 2, has been used as the material for the electrodes and contacts.

**Table 2:** The properties of electrodes^21^.

Electrical conductivity (S/m)	Relative permittivity	Coefficient of thermal expansion (1/K)	Heat capacity at constant pressure *J*/(*kg*.*K*)	Density $({\boldsymbol{k}}{\boldsymbol{g}}{\boldsymbol{/}}{{\boldsymbol{m}}}^{{\boldsymbol{3}}})$	Thermal conductivity ${\boldsymbol{w}}{\boldsymbol{/}}({\boldsymbol{m}}{\boldsymbol{.}}{\boldsymbol{K}})$	Young’s modulus (Pa)	Poisson’s ratio
4.56 × 10^6^	10000	14.2 × 10^−6^	129	19300	317	70 × 10^9^	0.44

#### Al_2_O_3_ substrate

The dimensions of alumina substrate that has been modeled in this work is 0.1 × 1 × 2 mm. The simulation showed almost the same results for the 1.5–2.5 mm substrate heights. Table 3 shows the properties of the substrate, used for the simulation.

**Table 3:** Properties of Al_2_O_3_ substrate^12,22^.

width (μm)	length (μm)	thickness (μm)	Electrical conductivity (S/m)	Relative permittivity	Coefficient of thermal expansion (1/K)	Heat capacity at constant pressure *J*/(*kg*.*K*)	Density $({\boldsymbol{k}}{\boldsymbol{g}}{\boldsymbol{/}}{{\boldsymbol{m}}}^{{\boldsymbol{3}}})$	Thermal conductivity ${\boldsymbol{w}}{\boldsymbol{/}}({\boldsymbol{m}}{\boldsymbol{.}}{\boldsymbol{K}})$	Young’s modulus (Pa)
1000	2000	100	0	5.7	605 × 10^−6^	730	3965	35	400 × 10^9^

#### The Heater

**Figure 13:**
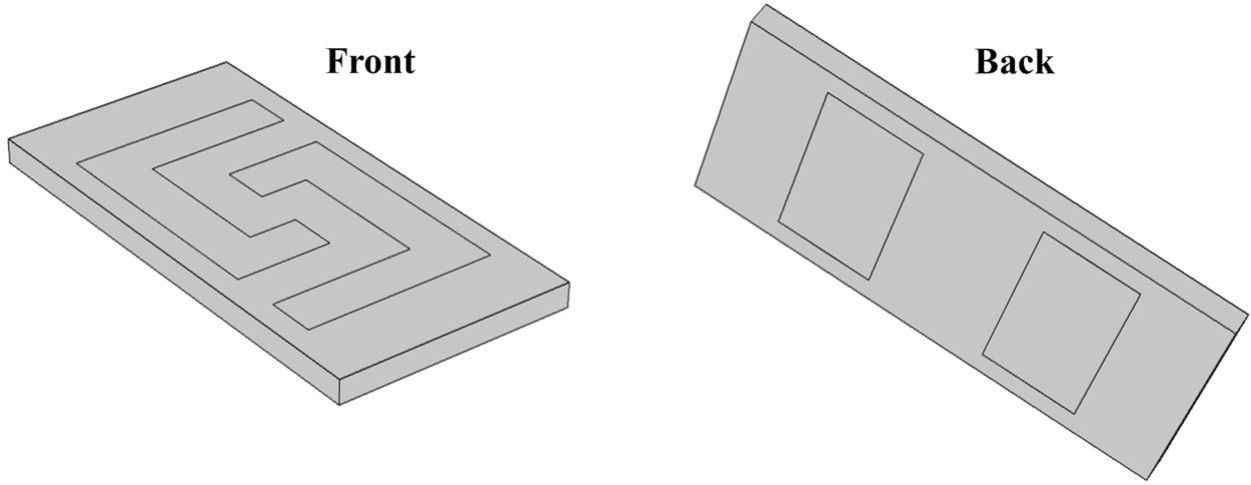
The structure of the sensor.

To model the heating element, RuO_2_ was placed between the gold contacts in an area of 800 × 500 µm^2^. Table 4 shows the properties of the Ruthenium dioxide used for the simulation. Figure 13 shows the whole structure of the sensor.

**Table 4:** Properties of RuO_2_ used in COMSOL^11,23^.

Electrical conductivity (S/m)	Thermal conductivity ${\boldsymbol{w}}{\boldsymbol{/}}({\boldsymbol{m}}{\boldsymbol{.}}{\boldsymbol{K}})$	Density $({\boldsymbol{k}}{\boldsymbol{g}}{\boldsymbol{/}}{{\boldsymbol{m}}}^{{\boldsymbol{3}}})$	Heat capacity at constant pressure (*J*/(*kg*.*K*))	Electrical resistivity (Ω.cm)
2.86	50	7050	0.42 × 10^−3^	35

### Mesh Study of the Model

**Figure 14:**
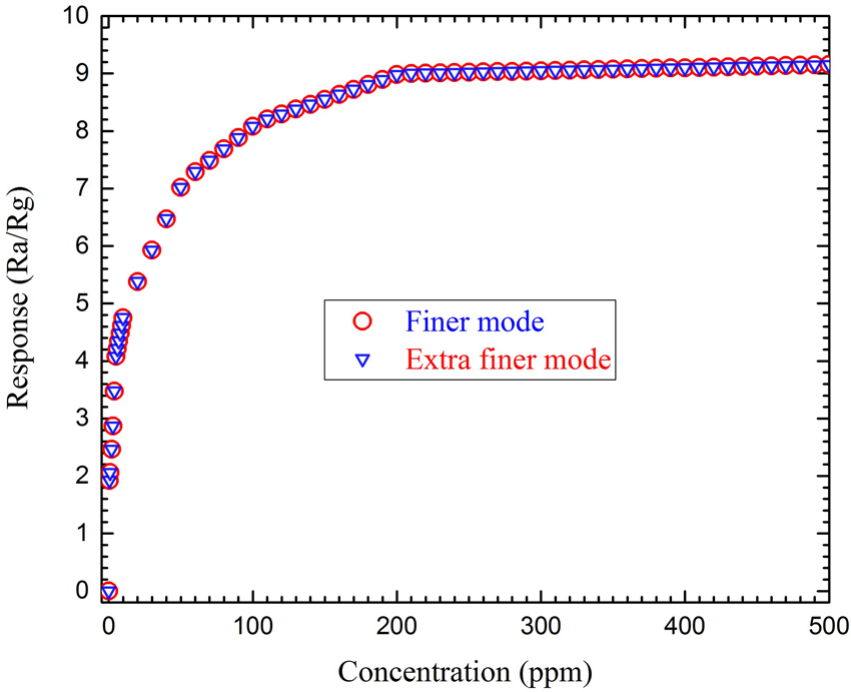
The response-concentration curve of the simulated sensor exposed to 0–500 ppm NO_2_ at finer and extra finer modes.

The mesh size for the whole structure was defined and optimized using finer mode in the COMSOL. The total 1,131,440 number of elements in the mesh structure was prefund in the fine mesh. If the mesh is defined lower than the finer mode, the results would be corrupted and if the mesh is defined denser, the results will have no changes with the input alterations. Figure 14 shows response-concentration graph of the sensor, plotted for both fine and extra fine modes.

## Conclusion

Analytical evaluation is very useful to determine general properties of a gas sensor, by measuring performance under input parameter variations, understood from experimental work. Simulation of a gas sensor allows a variable degree of abstraction, from simple to detailed, depending on the model built and of the corresponding parameters by the sensor. It also allows one to gracefully increase the sensor’s degree of detail such as the physical aspects and the composition of the materials involved at the sensing layer, such that the simulation development process can result in very close to the results of experiments under preformed conditions. In this work, a gas sensor based on the SnO_2_/rGO for detection of NO_2_ was modeled and simulated in COMSOL for the first time and its results were validated by comparing with a results of a fabricated sensor with same aspects and features. The results from the simulated sensor show a good agreement with the fabricated sensor with some inconsistencies due to differences between the practical conditions in the real chamber and applied conditions to the analytical equations such as the flow of the molecules of the applied gas and their collisions to the surface of the active layer in the real work, and the uniformity of the heat distribution in the simulated results. It was found out that the most assorted challenge for this kind of simulation that needs to be taken into account is the influence of temperature’s function on the simulation procedure which might be resulted in some degrees of incompatibility between the experimental and simulation results. Despite the inconsistencies, the presented model in this study is potentially capable to be developed for diversity of the target gases and can be employed with different types of active layer, sizes, and materials.
